# Genomic markers of midostaurin drug sensitivity in FLT3 mutated and FLT3 wild-type acute myeloid leukemia patients

**DOI:** 10.18632/oncotarget.27656

**Published:** 2020-07-21

**Authors:** Mara W. Rosenberg, Kevin Watanabe-Smith, Jeffrey W. Tyner, Cristina E. Tognon, Brian J. Druker, Uma Borate

**Affiliations:** ^1^ Knight Cancer Institute, Oregon Health and Science University, Portland, OR, USA; ^2^ Division of Hematology and Medical Oncology, Oregon Health and Science University, Portland, OR, USA; ^3^ Howard Hughes Medical Institute, Portland, OR, USA; ^4^ Department of Cell, Developmental, and Cancer Biology, Oregon Health and Science University, Portland, OR, USA

**Keywords:** acute myeloid leukemia, drug sensitivity, midostaurin, drug resistance, FLT3

## Abstract

Acute myeloid leukemia (AML) is a heterogeneous malignancy with the most common genomic alterations in NPM1, DNMT3A, and FLT3. Midostaurin was the first FLT3 inhibitor FDA approved for AML and is standard of care for FLT3 mutant patients undergoing induction chemotherapy [1, 2]. As there is a spectrum of response, we hypothesized that biological factors beyond FLT3 could play a role in drug sensitivity and that select FLT3-ITD negative samples may also demonstrate sensitivity. Thus, we aimed to identify features that would predict response to midostaurin in FLT3 mutant and wild-type samples.

We performed an *ex vivo* drug sensitivity screen on primary and relapsed AML samples with corresponding targeted sequencing and RNA sequencing. We observed a correlation between FLT3-ITD mutations and midostaurin sensitivity as expected and observed KRAS and TP53 mutations correlating with midostaurin resistance in FLT3-ITD negative samples. Further, we identified genes differentially expressed in sensitive vs. resistant samples independent of FLT3-ITD status. Within FLT3-ITD mutant samples, over-expression of RGL4, oncogene and regulator of the Ras-Raf-MEK-ERK cascade, distinguished resistant from sensitive samples. Overall, this study highlights the complexity underlying midostaurin response. And, our results suggest that therapies that target both FLT3 and MAPK/ERK signaling may help circumvent some cases of resistance.

## INTRODUCTION

Acute Myeloid Leukemia (AML) is a heterogeneous malignancy, most commonly affecting individuals ≥60 years of age [3, 4]. Technological and molecular advances have led to further classification and stratification of this disease by cytogenetic and mutational features, as well as the eventual development of many targeted therapies. A subtype of AML, classified by the presence of a FLT3-Internal Tandem Duplication (ITD) mutation, tends to have a worse prognosis with early relapse and death [5]. FLT3 is a class III receptor tyrosine kinase, most often expressed in hematopoietic stem, progenitor, and dendritic cells. Of particular interest in myeloid malignancies, FLT3 plays an important role in hematopoietic proliferation, differentiation and survival. ITD mutations in FLT3 occur within the juxtamembrane domain and lead to constitutive receptor activation [6].

FLT3 mutations occur in approximately 30% of *de novo* AML cases, of which, 25% are ITD mutations and 5% are tyrosine kinase domain (TKD) point mutations [7–9]. Per the 2017 European Leukemia Network (ELN) Guidelines, a complete diagnostic work-up should include screening for the presence of FLT3 mutations, as well as mutant-to-wild-type allelic ratios. A low allelic ratio is <0.5 while a high allelic ratio is >0.5, and allows for appropriate stratification into favorable, intermediate, or adverse risk classifications [10, 11]. With the increasing attention and importance that has been placed on FLT3 mutant AML, many targeted therapies have been designed to combat it [4, 8]. There are many past and present clinical trials examining the activity of tyrosine kinase inhibitors (TKIs) against FLT3 mutant AML, including sunitinib, midostaurin, lestaurtinib, sorafenib, ponatinib, crenolanib, gilteritinib, and quizartinib [8, 12–18].

Among the FLT3 inhibitors, midostaurin was the first to receive FDA approval and has FLT3-ITD and FLT3-TKD activity and also acts as a multi-kinase inhibitor. When combined with standard induction therapy, midostaurin provides a successful overall survival rate of 51.4% compared to 44.3% in placebo [2]. Despite an overall survival benefit, only 59% to 80% of patients treated with midostaurin achieve complete remission (CR)/complete remission with incomplete hematological recovery (CRi) with a fraction of these continuing on to develop resistance [19–21]. Thus, while patients with FLT3 mutations have improved outcomes, both primary and secondary resistance remains unfortunately still common. Factors predictive of the development of resistance include the initial presence of multiple leukemic clones, low FLT3-mutant allelic ratio, or additional primary mutations in the FLT3 kinase domain [5, 8, 11, 22].

We hypothesized that there are additional genomic alterations and gene expression changes outside of FLT3-ITD mutations that can influence AML sample resistance or sensitivity to midostaurin and aimed to further characterize these factors. Here, we provide evidence from an *ex vivo* drug sensitivity screen to suggest that KRAS or TP53 mutant samples have greater resistance to midostaurin as do FLT3-ITD mutant samples with RGL4 overexpression. Further, independent of FLT3 status, we identify a distinct gene signature correlating with midostaurin sensitivity.

## RESULTS

### Cohort

To understand the impact of different genomic alterations on midostaurin response, we identified a cohort of 193 Primary and 21 Relapse AML samples from the Beat AML published dataset [23]. This corresponds to 214 patients that were functionally assessed with midostaurin and annotated for FLT3 status. Unique samples per patient were chosen by prioritizing samples extracted from bone marrow aspirates over peripheral blood extractions and primary over relapsed disease status at the time of sample collection. Ultimately, 59% of samples were from bone marrow aspirates, 38% from peripheral blood, and 3% from leukapheresis (Supplementary Table 1). Median age was 61 years (interquartile range 44–71), with 52% male and 48% female (Table 1). Within this group, 73 samples were favorable risk, 59 samples intermediate, and 68 were adverse based on the 2017 ELN risk groups. Further, 12 samples did not have available data for their FLT3-ITD allelic ratio and thus were classified as having an indeterminate ELN risk. We found the commonly mutated genes NPM1, FLT3-ITD, FLT3-TKD, and DNMT3A to be mutated at 33%, 23%, 7%, and 16% in our cohort, respectively, consistent with previously reported prevalence [24].

**Table 1:** Patient characteristics

Characteristic	Count	Percent
**No.**	214	
**Type**		
Primary	193	90%
Relapse	21	10%
**Gender**		
Male	111	52%
Female	102	48%
**Age at diagnosis**	61.2 (44 - 71)	
**White blood cell count**	33.4 (12.8 - 69.9)	
**Percent blasts blood**	49.5 (20.8 - 80.0)	
**ELN Risk**		
Favorable	73	34%
Intermediate	59	28%
Adverse	68	32%
Indeterminate	12	6%
**NCCN Karyotype Risk**		
Better-risk	20	9%
Intermediate-risk	155	72%
Poor-risk	39	18%
**Fusions**		
CBFB-MYH11; inv (16)(p13q22)	15	7%
MLLT3-KMT2A; t (9;11)(p21; q23)	8	4%
RUNX1-RUNX1T1; t (8;21)(q22; q22)	7	3%
RPN-EVI1; inv (3)(q21q26.2)	4	2%
**Gene Mutations**		
NPM1	70	33%
FLT3-ITD	50	23%
DNMT3A	34	16%
NRAS	28	13%
CEBPA	24	11%
TET2	22	10%
IDH2	21	10%
ASXL1	18	8%
SRSF2	16	7%
WT1	16	7%
FLT3-D835	14	7%
KMT2A	14	7%
PTPN11	13	6%
RUNX1	12	6%
TP53	12	6%
KRAS	12	6%
IDH1	11	5%

### Mutation analysis

**Figure 1:**
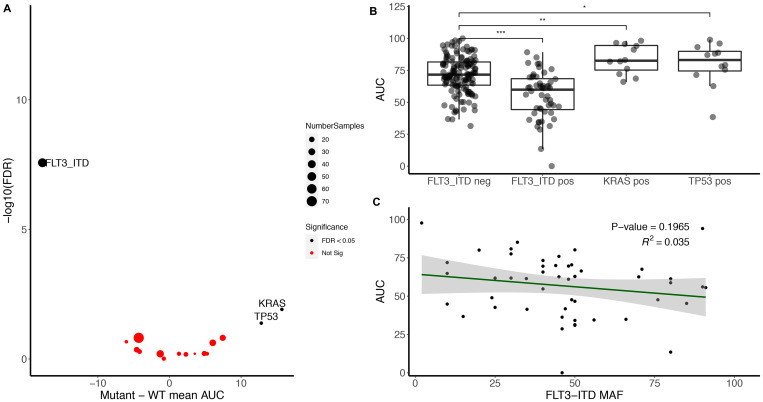
FLT3-ITD associates with midostaurin sensitivity while KRAS and TP53 mutations associate with midostaurin resistance. (**A**) Volcano plot representing the difference between mutant and wild-type midostaurin AUC for each gene present in at least 5% of the samples (Number of genes = 17; Number of mutant samples within that gene is annotated by circle size). Significance was calculated using Kruskal–Wallis H test and false discovery rate was used to correct for multiple hypothesis testing. (**B**) FLT3-ITD, KRAS, and TP53 mutant samples compared to FLT3-ITD negative cohort. Significance determined by Kruskal–Wallis (^***^, ^**^, and ^*^represent < 0.001, < 0.01, and < 0.05, respectively). (**C**) FLT3-ITD minor allele frequency compared to midostaurin AUC. Linear regression R-squared of 0.035, negative slope of 0.17 (*p* > 0.05).

To assess the impact of somatic mutations on midostaurin sensitivity, we compared all somatic alterations present in at least five percent of the samples. As expected we identified an increase in midostaurin sensitivity in FLT3-ITD positive patients compared with FLT3-ITD wild-type (Figure 1A and 1B). This is consistent with midostaurin’s mechanism of action as a FLT3 inhibitor [1]. Within FLT3-ITD mutations we did not observe a strong association between FLT3-ITD allele frequency and midostaurin AUC values via a continuous analysis (Figure 1C) though did observe a small but significant difference when split by the commonly used allelic ratio of 0.7 (*p* < 0.05) (Supplementary Figure 1) [2]. Mutated FLT3-ITD samples had a median AUC of 59.8 while non-mutated had an AUC of 73.2. The full dataset had a median AUC of 69.9 (IQR 48.0 to 91.8 with full range of 0 to 100). Further, we did not see an association of drug sensitivity to FLT3-TKD mutations (*N* = 14; Supplementary Figure 2).

Outside of FLT3-ITD, we observed an increase in resistance to midostaurin for samples with pathogenic KRAS and TP53 mutations (Figure 1A, right side). In contrast, NRAS was not significantly associated with an increase in resistance (Supplementary Table 2). When excluding FLT3-ITD from the cohort, we still saw an increase in drug resistance within samples with KRAS and TP53 mutations compared to non-mutated samples. There was an increase of median AUC from 72.9 to 82.5 for KRAS and 71.5 to 87.0 for mutated TP53 (Figure 1B). However, there was no correlation between allelic frequency and degree of midostaurin response (Supplementary Figure 3). To confirm the correlation of these mutations to midostaurin response, we identified an independent cohort of 43 FLT3-ITD negative samples collected under the Beat AML protocol however outside the initial data freeze. Nine were KRAS mutant, 34 KRAS wild-type, 11 TP53 mutant and 31 TP53 wild-type (one had unknown TP53 status). Given the expected variance in the dataset and difference between cohorts, we were powered at 80% to detect a difference in KRAS AUC and saw a similar trend towards increased drug resistance in KRAS mutant samples (*p =* 0.09, Supplementary Figure 4). There was no association found with TP53 in these samples.

### RNA-Seq analysis

**Figure 2:**
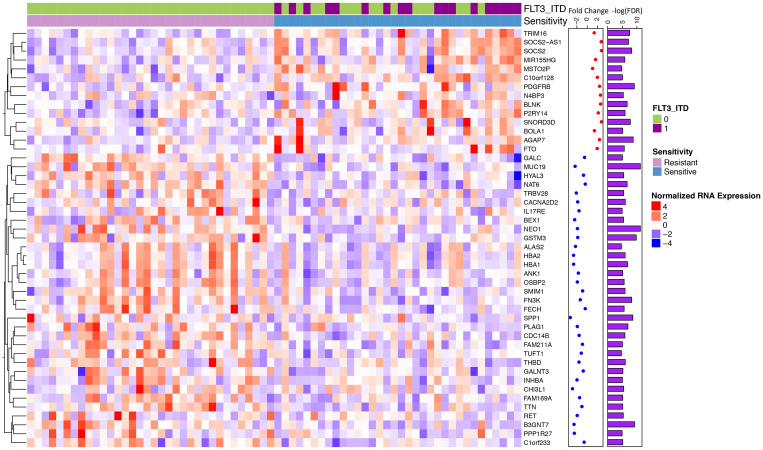
Differential gene expression for midostaurin sensitive vs. resistant samples identifies a unique signature. Normalized RNA expression for midostaurin sensitive (< 20th quartile AUC) and resistant (> 80th quartile) samples (34 sensitive, 34 resistant). Significantly differentially expressed genes shown (*N* = 47, FDR < 0.01). Overexpressed genes are shown by shades of red with under expressed genes by shades of blue. Fold change calculated between the two cohorts is annotated; with red representing those overexpressed in the sensitive compared to the resistant cohort and blue those that are under expressed.

Next, we sought to evaluate the impact of gene expression on drug sensitivity. We selected all *de novo* primary AML samples (*N* = 193) with RNA-Sequencing which provided a cohort of 170 patients of both FLT3-ITD mutated and non-mutated samples. We identified 47 differentially expressed genes between sensitive and resistance cohorts (false discovery rate corrected *p* < 0.01, See methods; Figure 2). Sensitive samples were defined as those samples below the 20th percentile of AUC (*N* = 34) and resistance samples were samples above the 80th percentile (*N* = 34). Midostaurin sensitive samples were enriched for FLT3-ITD mutant samples, however there remained 16 / 34 sensitive samples that were FLT3-ITD wild-type.

**Figure 3:**
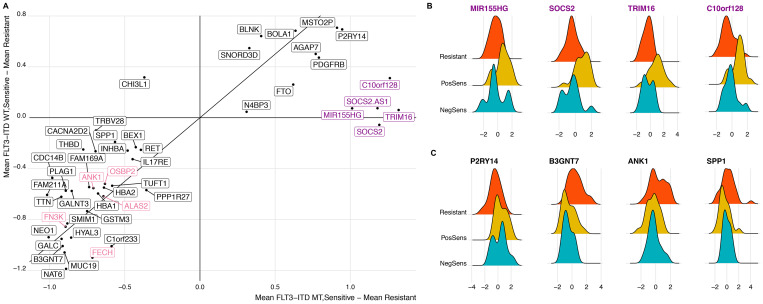
Distinct differential gene expression signature correlates with midostaurin expression regardless of FLT3-ITD status. (**A**) Scatter plot comparing all significantly expressed genes (*N* = 47). X-axis is calculated as the difference in the mean gene expression between sensitive and resistant samples within the FLT3-ITD mutant cohort. Y-axis displays the difference in mean gene expression between sensitive and resistant samples within the FLT3-ITD wild-type cohort. Highlighted are genes enriched in heme-metabolism and those known to associate with FLT3 status. (**B** and **C**) Distribution of midosaturin AUC between midostaurin-resistant, FLT3-ITD positive midostaurin-sensitive, and FLT3-ITD negative midostaurin-sensitive cohorts. Genes included are representative of those known to associate with FLT3-ITD status (B) and those independent of FLT3-ITD status (C).

Differentially expressed genes can be interpreted in two groups: those that have a change in expression in samples that are both mutant FLT3-ITD and sensitive and those that have a change between sensitive and resistant regardless of FLT3-ITD status. Within the FLT3-ITD mutated, sensitive cohort we observe known genes found to be over-expressed in FLT3-ITD mutated samples including SOCS2, TRIM16, MIR155HG, and C10orf128 (Figure 3A and 3B) [25–30]. However, we also observed a number of genes that had significant differential expression between sensitive and resistant cohorts that was not dependent on FLT3 status (Figure 3C). We found enrichment for overexpression of genes involved in heme metabolism in the resistant cohort (FDR corrected *p =* 0.0125) [31]. Additional genes that were overexpressed in the resistant samples (down regulated in the sensitive cohort) included those related to growth and mobility such as B3GNT7 known to be involved in cell migration and invasion and SPP1 (encoding for osteopontin) which is both a marker of poor survival in AML and related to adhesion, stemness, and differentiation [32–34]. Further, ANK1, a gene involved in erythropoiesis is known to be under-expressed in FLT3-ITD mutated samples, was downregulated in the midostaurin sensitive cohort regardless of mutation status [35] (Figure 3A).

We also identified genes that were upregulated in the midostaurin sensitive samples regardless of FLT3-ITD status. P2RY14, a G protein-coupled receptor, is part of the PI3K/mTOR pathway downstream of FLT3 suggesting a possible signaling event related to FLT3 activation targetable by midosaturin aside from FLT3-ITD mutations [15, 36].

**Figure 4:**
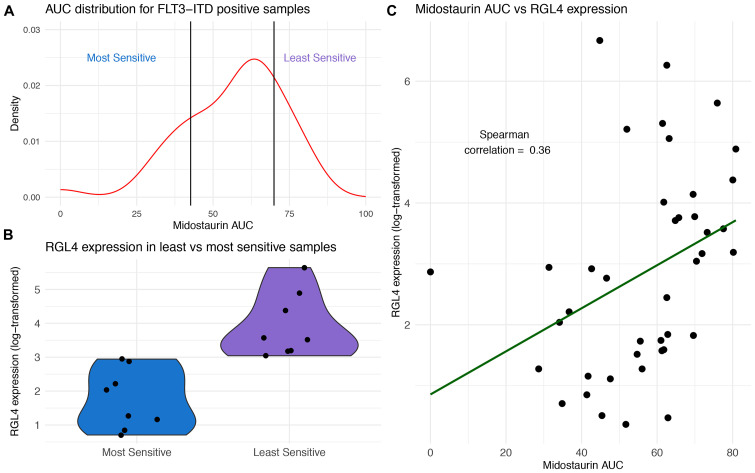
RGL4 expression correlates with response to midostaurin in FLT3-ITD positive samples. (**A**) Distribution of midostaurin AUC for FLT3-ITD positive samples with breakpoints for most and least sensitive set at the 20th and 80th AUC percentile, respectively (*N* = 41). (**B**) Violin plots of RGL4 expression in midostaurin sensitive and least sensitive samples. (**C**) Positive correlation (Spearman rho = 0.36) between midostaurin AUC and RGL4 expression across all FLT3-ITD positive samples (*N* = 41).

Further, within the FLT3-ITD positive samples, there was a range of responses with AUC values ranging from 0 to 89.2 (median 59.9, IQR 35.7–84.1). We sought to identify expression changes within the mutant cohort that stratified the patient by midostaurin response (Figure 4A). Performing differential gene expression, we identified RGL4 to be over-expressed in the FLT3-ITD positive, midostaurin-resistant cohort, and further showed that there was a positive correlation between AUC and RGL4 expression (Figure 4B and 4C). RGL4 (ral guanine nucleotide dissociation stimulator like 4) encodes for a guanine nucleotide exchange factor similar to Ral which causes activation of the downstream Ras-Raf-MEK-ERK pathway [37].

To confirm these findings, RGL4 expression was measured in 21 FLT3-ITD positive independent samples. Indeed, RGL4 over-expression within these samples correlated with increased midostaurin resistance (Supplementary Figure 5).

## DISCUSSION

Our research explored the multi-targeted nature of midostaurin and suggested a number of molecular mutational patterns that correlated with midostaurin drug sensitivity and resistance in both FLT3-ITD mutated and FLT3-ITD wild-type AML patient samples. In line with its known mechanism and previous reports, we observed that FLT3-ITD mutated patients had increased sensitivity to midostaurin though we did not identify an association with FLT3-TKD possibly due to the limited number of FLT3-TKD samples in our cohort. Further, we noted specific point mutations and gene expression patterns that may better explain the range of response to midostaurin treatment.

Within the FLT3-ITD positive cohort, an increased expression of RGL4, an oncogene and upstream regulator of the Ras-Raf-MEK-ERK cascade, correlated with a decrease in midostaurin response. Additionally, within the FLT3-ITD negative cohort, KRAS mutations correlated with a poorer midostaurin response. We did not have sufficient samples to investigate the impact of KRAS within FLT3-ITD AML. Leveraging the Genomics of Drug Sensitivity database (https://www.cancerrxgene.org/), RAS mutations were also seen to confer resistance in AML cell lines with NRAS mutations significantly mutated in midostaurin resistant cell lines (p = 0.003) [38] Combined, these findings suggest the involvement of Ras-Raf-MEK-ERK pathway as a possible escape mechanism for midostaurin therapy. RAS mutations as an escape mechanism have been established in the literature for other FLT3 inhibitors as well as additional small molecule inhibitors. Both KRAS and NRAS mutations have been shown to correlate with quizartinib resistance [39, 40] and NRAS with gilteritinib resistance [39, 40]. Further, co-occurring mutations in NRAS confer resistance to the IDH2 inhibitor enasidenib and activation of the RAS pathway is an escape mechanism for venetoclax [41, 42]. Here, we add the increased expression of RGL4 as a contributor to the RAS pathway of resistance. Additional functional studies are necessary to further elucidate this mechanism of resistance. Moreover, the impact of RGL4 expression as a primary and secondary resistance mechanism should be further investigated in larger retrospective cohorts or prospective studies.

Within our cohort we initially suggested TP53 as a marker of resistance, however were unable to confirm such correlation in a similar demographic validation set. This may have been due to a smaller effect size or larger variance than initially predicted. Additionally, given the limited number of samples in the validation set, there may have been non-annotated characteristics that influenced the results. To further investigate this mutation, we identified TP53 mutations as significantly mutated in midostaurin resistant AML cell lines (*p =* 0.0335) from the Genomics of Drug Sensitivity database [38]. It would be interesting to further explore this in a larger, independent patient cohort. Additionally, as TP53 is an established marker of adverse outcomes in AML and overall chemoresistance, it is likely that this resistance isn’t limited to midostaurin [43, 44].

We also observed that 16 / 34 of the most sensitive samples did not harbor a FLT3 mutation and a majority of differentially expressed genes were independent of FLT3 status. Given the non-specific nature of midostaurin, these patterns would suggest the efficacy of midostaurin outside of FLT3 mutant samples and highlights that additional biological factors, separate from presence or absence of FLT3 mutations, should be considered in predicting midostaurin response. Alternatively, these may also represent samples that are universally sensitive to a broad spectrum of inhibitors. However, additional functional studies are required to better characterize the mechanisms by which these mutations and expression patterns lead to the varied response patterns.

Midostaurin was the first new FDA approved agent for AML patients in over a decade and it is now standard of care to treat FLT3-ITD positive primary AML patients in combination with chemotherapy [45]. Alternative FLT3 inhibitors have been developed, with gilteritinib recently approved for treatment of adult patients with FLT3 mutated relapsed or refractory AML [12, 46]. With multiple FLT3 inhibitors available, it is important to understand the sensitivity mechanisms of each to better personalize therapy in chemo-refractory or relapsed patients. Here, we have leveraged an *ex vivo* drug sensitivity screen to propose sensitivity mechanisms based on individual mutations and gene expression patterns for both FLT3-ITD positive and FLT3-ITD wild-type samples. For FLT3-ITD positive samples, the increased numbers of patients currently being treated clinically with midostaurin will enable *in vivo* investigations of these suggested mechanisms, and, while not currently used clinically, these results might suggest the benefit of midostaurin in select FLT3-ITD wild-type patients as well.

Overall, we identify genomic alterations that correlate with midsotaurin response independent of FLT3-ITD status, propose that Ras-Raf-MEK-ERK inhibition in combination therapy could limit resistance to midostaurin, and suggest that within the overall AML population there may be therapeutic benefit of midostaurin in patients with certain expression profiles.

## MATERIALS AND METHODS

### Patients

All patients gave consent to participate in this study which leverages an existing dataset from the Beat AML cohort in addition to clinical targeted deep sequencing performed at Oregon Health & Science University (OHSU). Sample collection protocols received approval and guidance from the institutional review boards at OHSU, University of Utah, University of Texas Medical Center (UT Southwestern), Stanford University, University of Miami, University of Colorado, University of Florida, National Institutes of Health (NIH), Fox Chase Cancer Center and University of Kansas (KUMC).

Briefly, peripheral blood, bone marrow, and leukapheresis samples were extracted from all AML patients. Mononuclear cells (MNCs) were isolated by Ficoll gradient centrifugation and cell pellets were snap frozen in liquid nitrogen for subsequent DNA isolation (Qiagen, DNeasy Blood & Tissue Kit). Freshly pelleted cells were lysed immediately in GTC lysate for subsequent RNA isolation (Qiagen, RNeasy Mini Kit), and freshly isolated mononuclear cells were plated into *ex vivo* drug sensitivity assays within 24 hours of draw (described below).

Skin punch biopsies were collected at the site of Jamshidi needle insertion for subsequent bone marrow biopsies and genomic DNA was isolated for use as matched normal controls for exome sequencing (Qiagen, DNeasy Blood & Tissue Kit).

### Drug sensitivity screen

The extracted MNCs were exposed to escalating dose concentration gradients of small-molecule inhibitors – including midostaurin – at a concentration of 0.014, 0.041, 0.123, 0.37, 1.11, 3.33, and 10 μm. The cells were then incubated for 72 hours at 37° C in 5% CO2. Cell viability was then measured by determining the relative number of remaining MNCs *via* a tetrazolium-based colorimetric assay (CellTiter AQueous One Solution Cell Proliferation Assay; Promega, Madison, WI, USA).

### Custom gene panel (GeneTrails) sequencing and variant detection

Sequencing on a gene panel of 42 genes was performed as part of standard clinical care through the CLIA certified Cancer Diagnostics Laboratory at OHSU (GeneTrails). The custom capture panel of 42 genes is a set known to play a role in leukemia pathogenesis, prognosis, or response to therapy and include: ABL1, ASXL1, BCOR, CBL, CBLB, CEBPA, CREBBP, CSF3R, DNMT3A, ETV6, EZH2, FBXW7, FLT3, GATA1, GATA2, HRAS, IDH1, IDH2, IKZF1, IL7R, JAK1, JAK2, JAK3, KDM6A, KIT, KRAS, MPL, NOTCH1, NPM1 NRAS, PAX5, PTPN11, RUNX1, SF3B1, SRSF2, STAT3, SUZ12, TET2, TP53, U2AF1, WT1, and ZRSR2.

Genomic DNA was extracted and purified from blood or bone marrow, and sequenced by next-generation sequencing (NGS) using multiplexed PCR (AmpliSeq primers) and emulsion PCR, followed by semiconductor-based sequencing on an Ion Torrent PGM. Gene segments that were not easily covered by NGS are covered instead by Sanger dideoxy sequencing methods.

The minimum detection for the GeneTrails assay is 5% to 15% mutant allele fraction depending on sequence read depth, with a minimum sequence coverage depth of 100 ×.

### Exome sequencing and variant detection

A subset of samples within the Beat AML dataset possessed whole exome sequencing as well. In brief, Illumina Nextera RapidCapture Exome capture probes and protocol were used, giving coverage of 37 Mb of DNA coding regions. Libraries were run on a Hiseq protocol (2500 paired ends, 100 cycle) with five or six lanes per capture group [23].

For genotyping, AML paired/skin biopsies were realigned together and then somatic point mutations were identified with Mutect v1.1.7 and insertions/deletions were called using Varscan2 v2.4.1 [47, 48]. Further filtering for mutations in the paired samples, and in the samples that did not have a matched normal control, is described in the Beat AML cohort [23].

Mutations were combined with GeneTrails by prioritizing those calls identified by GeneTrails and then augmenting any samples without GeneTrails sequencing with the mutations identified by exome sequencing. While the Beat AML dataset included exome-wide mutations, this study focused on those genes mutated in at least 5% of the cohort.

### Internal FLT3-ITD and NPM1 mutation detection

Due to the challenge of identifying FLT3-ITD and the common NPM1 four-base pair insertion using the GeneTrails NGS protocol, FLT3-ITD and NPM1 mutation status was confirmed using an internally run PCR assay and capillary electrophoresis as described previously [23].

### Derivation of FLT3-ITD and NPM1 consensus calls

FLT3-ITD and NPM1 mutations identified using the internal capillary PCR test (described above) were prioritized over the CLIA/CAP laboratory sequencing (GeneTrails) when available. When GeneTrails results disagreed with the internal testing, samples underwent manual review.

### RNA sequencing

The Beat AML dataset was leveraged for RNA-Sequencing across a subset of the samples in the cohort. Briefly, RNA-Sequencing was performed using the Agilent SureSelect Strand-Specific RNA Library Preparation Kit, and sequenced on an Illumina HiSeq 2500 as described previously [23]. The final raw sequencing counts were then used for downstream analyses.

### RNA-Seq expression analysis

RNA-Seq analyses were postprocessed using EdgeR v3.7 [49]. Counts per transcript were normalized through conversion to counts per million (cpm). Transcripts were retained if they had values > 1 cpm in at least in 25 of the resistant and 25 of the sensitive samples. The greatest expression transcript per gene was chosen to represent the expression value of that gene. Trimmed mean of M-values (TMM) normalization was applied to account for compositional differences between libraries. Differential expression was performed using glmfit in EdgeR which uses a negative binomial generalized log-linear model to model the normalized read counts for each gene. Significance was determined with an alpha of 0.01 for FDR corrected p-values.

### *Ex vivo* functional drug screens

*Ex vivo* functional drug screens were performed on freshly isolated mononuclear cells from AML samples as previously described [50]. The tetrazolium-based colorimetric assay produced absorbance values (optical density) that were used to calculate cell viability. For each sample, the cells were incubated with inhibitors in a seven-dose dilution series (from 10 μM, at 1:3 ratio, to 0.014 μM) and viability on day three was normalized to the average all-kill well optical density in each plate. These normalized values were confined to a 0-100 range to produce a response variable that represented the percentage of the average control well viability.

Drug sensitivity was quantified as area under the curve (AUC) with the concentration on log-scale, and calculated via average cell viability across all concentrations. AUC was calculated as the area under the fitted probit curve (via direct integration) using all seven doses as x-values and cell viability with limits from 0 to 100% as the y-value, and then normalizing the AUC values to a 0 to 100 scale.

### Statistical analysis

Kruskal–Wallis *H* test was used to determine significance of differences in drug sensitivity measures between mutant and wild-type groups. *P*-values were corrected for multiple hypothesis testing using FDR. Statistical analysis was performed in R v.3.4.0.

## SUPPLEMENTARY MATERIALS



## Abbreviations

AML: acute myeloid leukemia
FLT3-ITD: FLT3-internal tandem duplication
TKI: tyrosine kinase inhibitors
ELN Guidelines: European Leukemia Network Guidelines
