# Supplementary material for: Genomic markers of midostaurin drug sensitivity in FLT3 mutated and FLT3 wild-type acute myeloid leukemia patients
Source: Oncotarget. 2020 Jul 21;11(29):2807–18. doi: 10.18632/oncotarget.27656 (PMC7381100; doi:10.18632/oncotarget.27656)
Supplement: Supplementary file 1 [file oncotarget-11-2807-s001.pdf]

Genomic markers of midostaurin drug sensitivity in FLT3 mutated and FLT3 wild-type acute myeloid leukemia patients

SUPPLEMENTARY MATERIALS

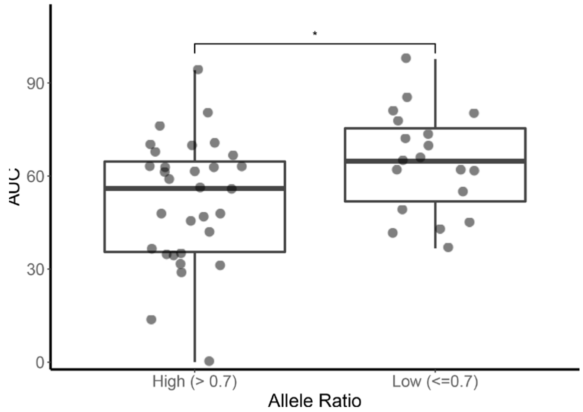

Supplementary Figure 1: Allele ratio of FLT3-ITD vs. midosaturin AUC. Comparison of FLT3-ITD allele ratio, calculated by mutant allele/reference allele to midostaurin AUC. Middle bar in the figure represents median AUC with edges as the 25th and 75th quartile. Significance calculated by Kruskal–Wallis *H* test with *representative of a *p*-value < 0.05.

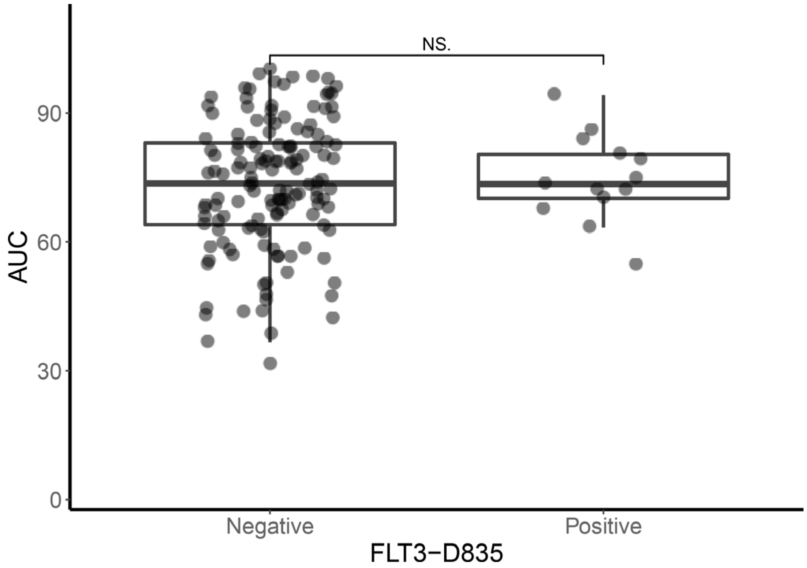

Supplementary Figure 2: Sensitivity to midostaurin is not significantly different between FLT3 wild-type and FLT3-D835 positive samples. Comparison of midostaurin AUC between FLT3 wild-type to FLT3-D835 (TKD) mutant samples. Middle bar in the figure represents median AUC with edges as the 25th and 75th quartile. Significance calculated by Kruskal–Wallis *H* test with NS representative of not significant.

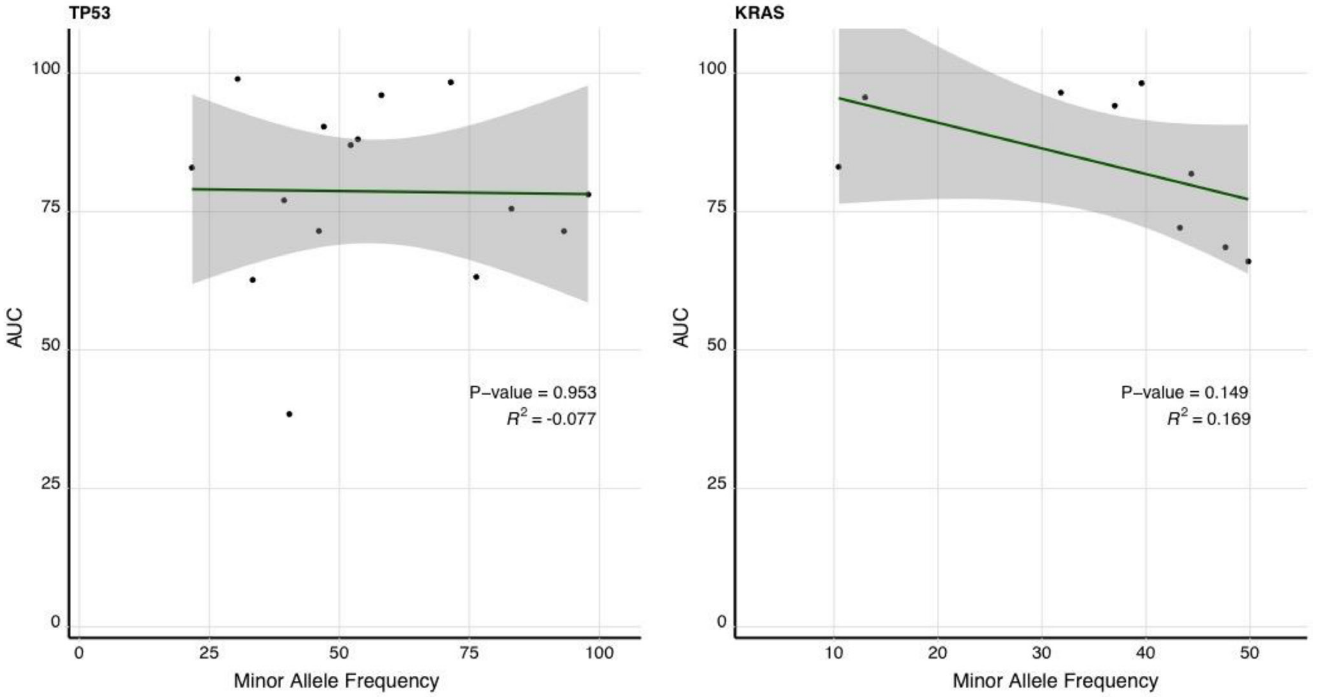

Supplementary Figure 3: There was no significant relationship between midostaurin sensitivity and KRAS or TP53 allele frequency. Comparison of the allelic frequency of TP53 or KRAS mutations to midostaurin AUC. *P*-values for the correlations were 0.953 and 0.149 for TP53 and KRAS respectively. Neither demonstrated significance.

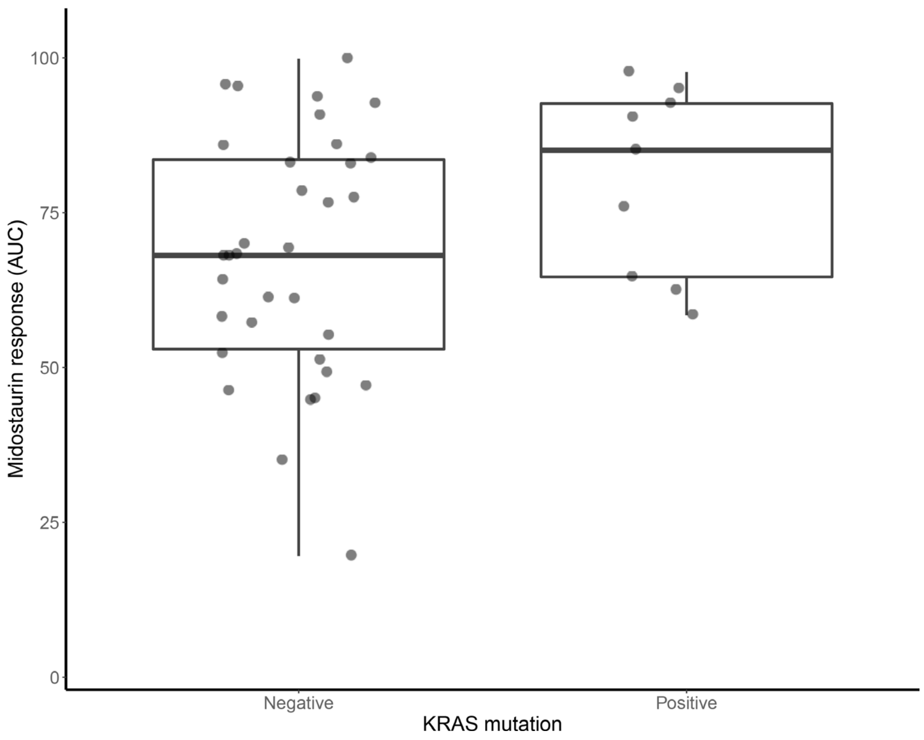

Supplementary Figure 4: Decreased midostaurin sensitivity in KRAS mutant vs. wild-type samples in independent samples. Comparison of Midostaurin AUC between KRAS mutant and wild-type samples within independent samples. Statistical significance ($p = 0.09$) calculated using Kruskal–Wallis test.

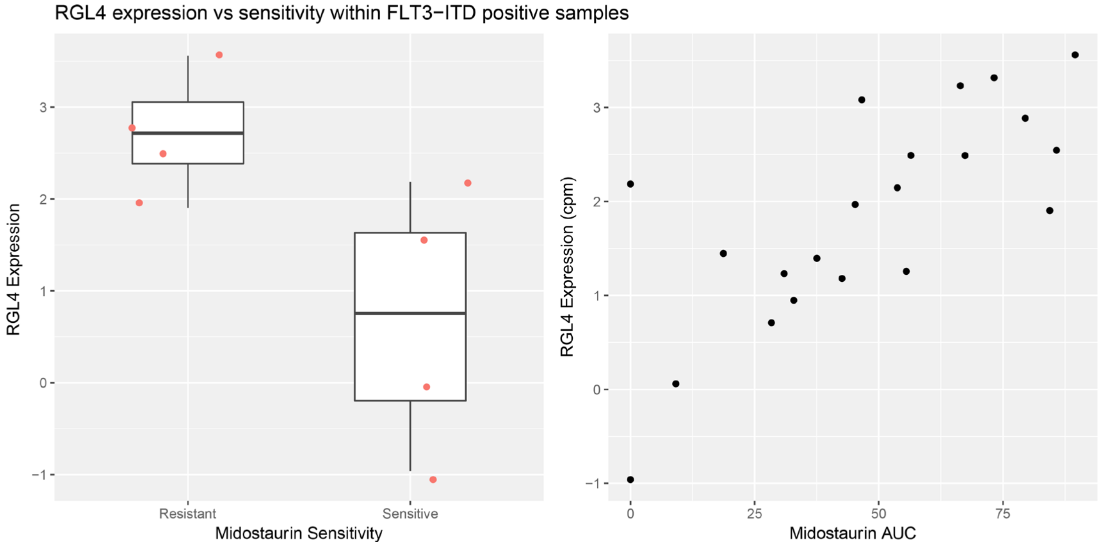

Supplementary Figure 5: RGL4 expression correlates with midostaurin sensitivity in independent FLT3-ITD mutant samples (*N* = 21). (**A**) Midostaurin sensitivity in the bottom least vs. top most sensitive samples within the cohort of 21 compared to RGL4 expression (measured as log10 of counts per million). Least and most sensitive samples were identified as FLT3-ITD mutant samples having a midostaurin AUC in the top 80th or bottom 20th from within the cohort of 21 ($N = 4$ respectively). (**B**) Midostaurin AUC across all FLT3-ITD mutant samples ($N = 21$) have positive correlation with RGL4 expression (spearman correlation +0.74).

Supplementary Table 1: Specimen type across the patient cohort

Specimen type	Number (*N*)	Percentage (%)
Bone Marrow Aspirate	126	59%
Peripheral Blood	82	38%
Leukapheresis	6	3%

Supplementary Table 2: Association of all investigated mutations with midostaurin sensitivity

Gene	Fold change	Mutated samples (*N*)	Significance
IDH1	3.5	11	Not Sig
KRAS	15.7	12	FDR < 0.05
TP53	12.8	12	FDR < 0.05
RUNX1	-6.0	12	Not Sig
PTPN11	5.3	13	Not Sig
FLT3_D835	1.3	14	Not Sig
KMT2A	-0.79	14	Not Sig
WT1	2.3	16	Not Sig
SRSF2	-4.2	16	Not Sig
ASXL1	4.9	18	Not Sig
IDH2	-4.6	21	Not Sig
TET2	-1.2	22	Not Sig
CEBPA	7.4	24	Not Sig
NRAS	6.0	28	Not Sig
DNMT3A	-1.3	34	Not Sig
FLT3_ITD	-17.7	52	FDR < 0.05
NPM1	-4.3	70	Not Sig
